# Evaluating the Effectiveness of the School-Based Sustainable Innovation for Children Transporting Actively Intervention: Protocol for an Age-Cohort Study

**DOI:** 10.2196/92946

**Published:** 2026-06-05

**Authors:** Hanna Forsberg, Anna-Karin Lindqvist, Stina Rutberg, Palma Chillón, Veronica Sjöberg, Annie Palstam

**Affiliations:** 1 Department of Health, Education and Technology Luleå University of Technology Luleå, Norrbotten Sweden; 2 Department of Physical Education and Sports Faculty of Sport Sciences, Sport and Health University Research Institute (iMUDS) Universidad de Granada Granada, Andalusia Spain; 3 School of Health and Welfare Dalarna University Falun, Dalarna Sweden; 4 Department of Clinical Neuroscience Institute of Neuroscience and Physiology, Shalgrenska Academy University of Gothenburg Gothenburg, Västra Götaland Sweden; 5 Rehabilitation Medicine Sahlgrenska University Hospital Gothenburg, Västra Götaland Sweden

**Keywords:** age cohort, active commuting, feasibility, physical activity, sustainability

## Abstract

**Background:**

Few children achieve the recommended daily levels of physical activity. Active school transportation (AST) offers a valuable opportunity to increase children’s physical activity. Initiatives promoting AST in early childhood are important and have the potential to reverse the decline in physical activity.

**Objective:**

This study protocol outlines an age-cohort design to evaluate the effectiveness of the Sustainable Innovation for Children Transporting Actively (SICTA) intervention on children’s AST.

**Methods:**

SICTA is a 4-week school-based intervention that incorporates gamification elements to enhance motivation and aims to increase AST in children. The intervention involves children and their parents as gatekeepers, as well as teachers delivering the intervention. All children, parents, and teachers in all schools, from grades 4 to 6 in one municipality in the southern part of Sweden, will be invited to participate in the evaluation. Following the age-cohort design, students at baseline (late fall 2024) will be compared with students of the same age from the same schools 1 year later at follow-up (late fall 2025) after receiving the intervention (implemented in late fall 2025). Using questionnaires at baseline and follow-up, this study will examine the effects of the intervention on children’s levels of AST. Children’s independent mobility and several mediators related to AST in both children and parents, based on the theory of planned behavior, the transtheoretical model of change, and self-determination theory, will also be examined. The intervention will also be evaluated from a sustainable perspective using the sustainable value equation. At follow-up, data collection will include a questionnaire for teachers assessing the feasibility of the intervention.

**Results:**

The project is funded for the period 2024-2026, and follow-up data collection is ongoing following completion of the intervention in late fall 2025. Results will be reported according to the predefined outcomes, including changes in AST, children’s independent mobility, psychosocial determinants among children and parents, and sustainability outcomes, as well as feasibility.

**Conclusions:**

The findings are expected to provide valuable insights into the effectiveness of the SICTA intervention, facilitating knowledge dissemination among end users and policymakers.

**Trial Registration:**

Swedish National Research Database Researchweb.org 283668; https://tinyurl.com/496rc6r4

**International Registered Report Identifier (IRRID):**

DERR1-10.2196/92946

## Introduction

### Background

Physical activity is essential for the well-being of children and adolescents [1]. Despite its importance, the majority of children and adolescents globally do not meet the recommended 60 minutes of physical activity per day [2]. In addition, disparities in physical activity levels and related health outcomes persist across populations, with children from less socially advantaged backgrounds being less likely to engage in regular physical activity [3,4]. This highlights the essential role of schools as a key setting for health promotion, providing equitable access to interventions that reach children across all socioeconomic backgrounds [5]. Furthermore, active school transportation (AST), typically involving walking or cycling for all or part of the transport to and from school, can make a significant contribution to a child’s total daily physical activity [6,7]. Additionally, previous research has suggested that AST is positively associated with cardiovascular fitness, academic performance, safer traffic environments, reduced air pollution, and lower greenhouse gas emissions [8-11]. In addition, children’s independent mobility (CIM), defined as the ability of children to move around freely without adult supervision, has been shown in previous studies to be an important facilitator of increased physical activity and AST [12-14]. Thus, promoting AST and CIM has the potential to address important societal challenges and contribute to several Sustainable Development Goals (SDGs) in the United Nations 2030 Agenda [15]. However, in many Western countries, there has been a marked decline in the prevalence of AST as well as CIM during the last few decades [16-19]. Nevertheless, recent trends indicate that the decrease might be leveling off somewhat [20]. However, there is still great potential to increase AST as a means of promoting physical activity levels in children [20]. Additionally, as physical activity behaviors, such as AST, are often established in childhood and persist into adulthood, health-promoting initiatives should be introduced early in life [21,22]. Moreover, although several initiatives have been launched to address AST, evidence-based and scalable solutions remain scarce. Systematic reviews indicate that interventions can increase AST among children and adolescents; however, the studies vary widely, and the quality of evidence is generally low [23,24]. High-quality research using behavior change theories as a basis for intervention design, as well as robust evaluation methods and reliable measurement instruments, is needed to better understand AST and assess intervention effectiveness.

### Sustainable Innovation for Children Transporting Actively: Development of the Intervention

The research project Sustainable Innovation for Children Transporting Actively (SICTA) was formed as a 4-week school-based intervention initially developed in 2016 in Sweden [25]. The SICTA intervention is based on theory, with 3 main concepts: social cognitive theory, empowerment, and gamification. Social cognitive theory involves factors related to AST behavior, including knowledge, self-efficacy, outcome expectations, short- and long-term goals, facilitators, and impediments to change. Moreover, empowerment facilitates the involvement of end users—children, teachers, and parents—in outlining the intervention. Gamification, which is the use of game design in a nongame context, is used to motivate children to participate in the intervention and perform assignments that are applicable to their route to school. The assignments are integrated into the curriculum; for example, one assignment could involve counting people and animals encountered during AST and analyzing these data in math classes. A long-term goal is also to track the number of kilometers walked and cycled by the class during the project. Thus, in line with the idea of gamification, when completing assignments, children receive rewards when they are accomplished. The intervention consists of 5 different steps: engaging parents through a parental meeting, increasing children’s knowledge of active transport, preparing weekly assignments for the children, encouraging walking and cycling to school over 4 weeks, and finally celebrating their achievements. At the parental meeting, parents are provided with information about the intervention, its benefits for AST, and an opportunity to discuss any concerns or perceived risks. To support teachers, they have access to an online platform called the Teacher’s portal. The assignments developed in the portal use innovative, game-based, student-centered pedagogical approaches that are integrated into contemporary school curricula. The criteria used during the development process included ensuring that the activities had a scientific foundation in educational research, an explicit connection to syllabi, opportunities for student influence, and the incorporation of peer collaboration. Therefore, it is evident from the previous description that the SICTA intervention is complex, involving multiple components and requiring skills from both the deliverers and the recipients [26]. Its complexity also arises from the flexibility of the intervention’s components and delivery, as well as the dynamic interactions between the intervention, its context, parents, children, and teachers. This adaptability ensures alignment with each school’s unique structure and functionality. In addition, guidelines stress the importance of considering both the intervention’s components and their interactions with the environment [26].

### SICTA: Previous Research

Since its initial development, SICTA has been continuously refined and evaluated, primarily through qualitative studies that focus on the perspectives of children, parents, and teachers [27-31]. These studies revealed that the intervention has received positive feedback from children, teachers [27,29,30,32], and parents [28,31], and a long-term qualitative follow-up showed that it fostered a habit of using AST among participants [30]. In 2020, the SICTA research project advanced to the next phase, SICTA 2.0, and feasibility evaluation continued. Qualitative evaluations have shown that teachers find the intervention flexible, meaningful, and easy to implement [32]. To summarize, the research around this project has led to an understanding of three key values, encapsulated by the acronym “FIT,” for different groups of end users: (1) it is a *fun* intervention for the children, (2) it *inspires* peace of mind for the parents, and (3) it is *teacher friendly*.

Moreover, in 2022, a nonrandomized controlled pilot study was conducted to assess the feasibility of the evaluation design, with a mix of qualitative and quantitative data [33]. The results highlight the complexity and challenges of conducting controlled research among school children. Although children were positive about participation and found reporting to be easy, our results invoke the need to use alternative evaluation designs and recruitment strategies that attract children using all modes of travel when evaluating AST interventions in school contexts. Using schools as the intervention arena allows almost all children to be reached, addressing inequities in physical activity and health [34]. However, schools are complex environments where randomized controlled trials (RCTs) are challenging [35]. Our pilot study showed low participation willingness and dissatisfaction among control school children, suggesting that an RCT is not suitable for a full-scale evaluation [33]. However, an age-cohort design has been proposed as a more feasible approach for evaluating interventions within a school setting [35].

In summary, this study protocol outlines an age-cohort design [35] to evaluate the effectiveness of the SICTA intervention on children’s AST. It also involves evaluating the intervention from a sustainable perspective. More specifically, the study addresses the following research questions: (1) How does the school-based intervention SICTA influence children’s active transportation to and/or from school? (2) How does the intervention influence CIM? (3) Which psychosocial aspects and sociodemographic characteristics are related to AST among children and parents? and (4) Can a sustainable value equation be applied to assess the sustainable value of the intervention?

## Methods

### Study Design

**Figure 1:**
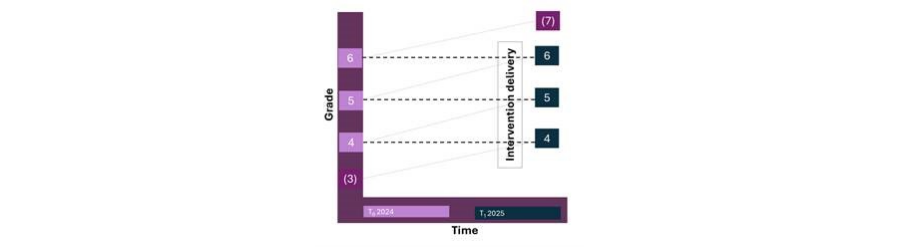
Visualization of the age-cohort design to compare students of the same age within the same schools over time, in relation to the Sustainable Innovation for Children Transporting Actively Intervention. T0 denotes baseline (2024), and T1 denotes follow-up (2025).

An age-cohort design will be applied to evaluate the effectiveness of the SICTA intervention on children’s AST [35]. In an age-cohort design, same-age students within the same schools are compared over time (Figure 1). For example, students in fifth grade at baseline (T_0_) will be compared with students in fifth grade from the same school at follow-up (T_1_) 1 year later. With such a study design, possible age-related maturational differences between the comparison groups are controlled. The SICTA intervention is evaluated using this age-cohort design, where students at baseline (late fall 2024) will be compared with same-aged students from the same schools 1 year later at follow-up (late fall 2025) after receiving the intervention (implemented in late fall 2025). The study was registered in the Swedish national research database Researchweb (project 283668) on October 25, 2024.

### Study Sample

In collaboration with the municipality involved in the project, public schools involving grades 4 to 6 will be identified, and invitations will be sent to principals to participate. Upon acceptance of participation by school principals, all children in grades 4 to 6, along with their parents and teachers at the participating schools, will be invited to participate in the study.

### Data Collection and Measurements

For all the research questions, data will be collected from the children and their parents at 2 time points, baseline (T_0_) and follow-up (T_1_). In addition, data will be collected from teachers at follow-up (T_1_). Multimedia Appendix 1 provides an overview of the data collection, including instruments used at baseline and follow-up. A detailed description of data collection and analysis is provided in the following sections for each research question. The baseline data collection is planned to be performed during 2 weeks in late fall 2024, and the data collection following the intervention is planned to be performed exactly 1 year later, 2 weeks after the 4-week SICTA intervention has ended, in line with the age-cohort design (Figure 1).

### Research Question 1

The number of weekly active school transports (0-10) will be collected using the wASTapp [36]. The wASTapp is a web-based form developed to enable daily self-reports of transportation mode, time, and distance and has shown satisfactory criterion validity when tested in children in grades 4 to 6. In the wASTapp web-based form, children are able to report their daily transport either in the classroom guided by their teachers (using school computers) or on their own phones. Children who report via their phone will receive a daily SMS text message containing a link to the web-based form. If children lack access to a school computer or mobile phone for completing self-reported questions, paper-based reporting will be provided as needed.

### Research Question 2

Data on CIM will be collected using the CIM License questionnaire [37]. The questionnaire consists of 6 questions with “yes” and “no” answer options and assesses the level of independent mobility in children.

### Research Question 3

To assess psychosocial aspects in children and parents, 2 questionnaires will be used: PILCAST (parents’ intention to let their child use active school transportation) [38] for parents and BRACS (behavioral regulation in active commuting to and from school)-Sweden [39] for children. PILCAST, based on the theory of planned behavior, includes 32 items on parents’ intentions, attitudes, norms, and perceived control and is assessed with 7-point Likert scales [38]. It also covers the transtheoretical model and sociodemographic characteristics such as gender, academic year, distance to school, and parents’ education level. BRACS-Sweden is based on self-determination theory and assesses children’s motivation to use AST using 23 items measuring intrinsic motivation, various types of regulation, and amotivation on a 5-point Likert scale [39]. It also includes sociodemographic details such as gender, distance to school, and academic year.

### Research Question 4

Sustainable health care aims to maintain or restore health while minimizing environmental harm and ensuring future generations’ health. The sustainable value equation [40], developed in health care research, will be used to analyze outcomes for patients and populations, considering financial, environmental, and social impacts [41].



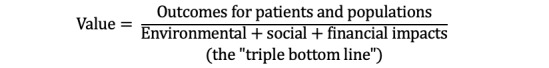



To evaluate the sustainability of the intervention, we will use data collected at baseline and following the intervention, applying the triple bottom line equation. Data on intervention outcomes are changes in AST, which will be retrieved from child reports in wASTapp and evaluated in relation to the environmental, social, and financial impacts of the intervention, as described in detail in the following sections.

### Data Collection on Environmental Impact

The environmental impact will be calculated on the basis of AST reports (wASTapp), where calculations will be conducted to evaluate changes in motorized transport, and the corresponding carbon dioxide emissions will be retrieved from standardized models provided by the Swedish Meteorological and Hydrological Institute [42].

### Data Collection on Social Impact

Social impact will be estimated using data obtained from self-reported questions on psychosocial aspects among the children (BRACS and CIM License) and parents (PILCAST) [37-39]. We will also collect data on social impact from teachers participating in the project using a questionnaire on aspects of the feasibility of the intervention, in accordance with the study by Bowen et al [43]. The questionnaire covers 5 domains: demand, acceptability, implementation, practicality, and limited efficacy. Demand represents documented use, while acceptability encompasses suitability, attractiveness, and reactions to the procedure. Implementation reflects the degree of execution, including aspects such as fidelity (the extent to which the intervention is delivered as intended) and dose (the amount of the intervention delivered or received). Practicality considers positive or negative effects and the feasibility of carrying out the procedure. Finally, limited efficacy indicates the intervention’s potential for success [43].

### Data Collection on Financial Impacts

The intervention costs are limited to the cost of the teachers for the time they devote to the intervention. Teachers delivering the intervention will be instructed to keep a record of time devoted to preparing, delivering, and following up on the intervention.

### Data Analysis

#### Quantitative Analysis

For all research questions, descriptive statistics will be used to summarize the data. Categorical variables will be reported as frequencies and percentages, and continuous variables will be reported as means and SDs or medians and IQRs, depending on distributional characteristics. Before the main analyses for research questions 1 to 3, the design effect will be evaluated to account for clustering within schools. Specifically, the intraclass correlation coefficient will be estimated for each outcome using an unconditional linear mixed-effects model with school as a random intercept [44]. For research questions 1 to 2, the data (wASTapp and CIM) are planned to be treated as continuous outcomes, as the wASTapp measure is based on a 0 to 10 scale and the CIM variable will be operationalized as an index ranging from 0 to 6, derived from 6 items. Changes from baseline (T_0_) to follow-up (T_1_) will be analyzed using an analysis of covariance, with T_1_ as the dependent variable and T_0_ included as a covariate. The models are planned to be adjusted for sociodemographic characteristics. For research question 3, associations between psychosocial factors, sociodemographic characteristics, and AST will be analyzed using analysis of covariance, with AST at follow-up (T_1_) as the dependent variable and baseline values (T_0_) included as a covariate. For all research questions (1-3), model assumptions will be assessed, and the final choice of regression model will be guided by the distribution of the outcomes. Although the outcomes are planned to be treated as continuous, alternative approaches (eg, ordinal models) will be considered if this is not supported by the data [45]. If clustering within schools is present, corresponding mixed-effects models will be applied. Missing data will be examined for extent and patterns [45]. If missing at random is considered plausible, multiple imputation will be used. Sensitivity analyses will include complete case analyses to assess robustness. A significance level of .05 will be applied. All analyses are planned to be conducted using SPSS (version 31; IBM Corp).

#### Sustainability Analysis

The analysis related to research question 4 will apply the sustainable value equation (the “triple bottom line”) and will be performed in collaboration with researchers at the Centre for Sustainable Healthcare, England. The analysis will include descriptive data on each aspect of the sustainable impact of the intervention in relation to the outcome. The analysis will not yield a single numerical result from the equation; rather, it will provide a transparent assessment of all sustainability components involved.

### Ethical Considerations

Ethical guidelines outlined in the World Medical Association’s Declaration of Helsinki will be adhered to throughout the study process. Information about the study will be provided to parents, children, and teachers in an appropriate format, explaining the purpose of the study, what participation entails, and that participation is voluntary. Informed consent will be obtained from parents or legal guardians prior to children’s participation. Children will be informed about the study and asked for their consent before participation. They will be made aware that they can decline participation or withdraw at any time without any consequences. Parents and teachers will provide consent for their participation. Participants’ privacy and confidentiality will be ensured throughout the study, and no identifiable personal information will be disclosed. Ethics approval for this study has been sought at the Swedish Ethical Review Authority, and the project was deemed to be exempt (Dnr: 2024-04069-01).

## Results

The project is funded for the period 2024-2026 and, follow-up data collection is ongoing following completion of the intervention in late fall 2025. Results will be reported according to the predefined outcomes, including changes in AST, CIM, psychosocial determinants among children and parents, and sustainability outcomes, as well as feasibility. Results will be presented using descriptive and inferential statistics as outlined in the analysis plan, and sustainability outcomes will be reported using a transparent assessment of environmental, social, and financial impacts.

## Discussion

### Expected Contributions

This study protocol outlines an age-cohort design to evaluate the effectiveness of the SICTA intervention on children’s AST. Multiple outcomes in children and parents will be analyzed, drawing on theoretical frameworks such as the theory of planned behavior, transtheoretical model of change, and self-determination theory. Additionally, this study will apply the sustainable value equation to evaluate the social, economic, and environmental sustainability of the intervention.

Our previous results highlight the complexity and challenges of conducting controlled research among school children [33]. Given these challenges, we have chosen an age-cohort design [35] to evaluate the SICTA intervention. This approach is particularly well suited for studying school-based interventions, as it allows for the examination of developmental changes over time while maintaining the feasibility needed for real-world implementation [46,47]. Age-cohort designs offer several advantages in this context. By following different age groups as they progress through school, this method enables a longitudinal perspective on intervention effects without requiring random assignment [35,47]. This not only improves practical feasibility but also minimizes issues such as ethical concerns, interference, and contamination—challenges often encountered in school-based studies [47]. In a previous pilot RCT of the SICTA intervention, significant participant attrition due to study design constraints further underscored the need for a more adaptable approach [33]. Additionally, age-cohort designs align well with the practice-based evidence framework, which is increasingly emphasized in public health research [46]. Many school-based interventions are implemented as natural experiments, making it essential to adopt methodologies that reflect real-world conditions rather than rigid experimental controls. By using an age-cohort design, we can capture broader trends within the school context while enhancing the generalizability of our findings [47]. However, given the pragmatic age-cohort design and real-world implementation, the findings should be interpreted as reflecting changes associated with the intervention rather than causal effects. In line with the Medical Research Council framework for complex interventions, effectiveness in this study refers to the extent to which the intervention may produce intended outcomes under real-world conditions, acknowledging contextual variability and implementation flexibility [26].

The SICTA intervention has been designed and piloted with a focus on feasibility and practical implementation within school contexts, as demonstrated in several research studies [27-32]. Building on social cognitive theory and incorporating the concepts of empowerment and gamification [25] has proven to be a solid foundation for success in previous research. Reviews on promoting AST in school settings emphasize the importance of using theoretical frameworks [24,48,49]. Our study also introduces a research-based, user-friendly digital teacher portal for promoting AST within the school context, which may contribute to the maintenance and sustainability of the intervention. To balance the need for rigorous research with the practical constraints of real-world school settings, some pragmatic adaptations will be made. These adaptations address mainly the implementation, considering the limited time and resources available in schools. On the basis of the key concept of empowerment and findings from a pilot study [32], it was evident that teachers prefer flexibility in the intervention’s layout. Therefore, they will have the freedom to choose the content and the number of assignments for students during the project period.

The primary goal of the SICTA intervention is to increase the number of participants using AST. However, evidence on the effectiveness of AST interventions in changing behaviors is mixed. For instance, Pang et al [49] reported that only half of the AST interventions reviewed reported positive behavior changes. Similarly, Schönbach et al [50] concluded that the evidence for school-based interventions promoting AST is insufficient, highlighting the early stage and methodological limitations of this research field. Therefore, it is essential to measure mediators to identify pathways for achieving future behavior changes, a step often overlooked in many AST intervention studies [48], as well as to recognize the complex nature of engagement in AST, which is shaped by environmental, social, and normative contexts. Our study protocol aims to identify several psychosocial factors related to AST behaviors among children and parents, as well as teachers’ perceptions of the feasibility of the intervention. In such efforts, we will use previously developed and validated instruments for both children and parents that have been adapted to the Swedish context [36,38,39]. The findings from the SICTA study will enhance current understanding of the effectiveness of a school-based intervention in promoting AST among primary school students.

### Implications

AST and CIM are essential for physical health, the development of social and cognitive skills, and preparing children for sustainable mobility choices in adulthood [21,22]. This study addresses low physical activity levels and the decline in active transport among children [2]. We anticipate that this school-based intervention will promote AST and CIM, ensuring accessibility to all children regardless of socioeconomic background, thereby contributing to improved health outcomes across all groups. If effective, the intervention could address public health challenges, improve academic performance in children, improve urban air quality, and contribute to climate change mitigation [8-11]. Furthermore, the unique assessment of the sustainable value of the intervention could contribute to cutting-edge knowledge for future public health policy. Thus, this study could contribute to several SDGs [15].

The World Health Organization–United Nations Children’s Fund–Lancet Commission highlights the enormous benefits of investing in children’s health, including nonmonetary gains such as enhanced citizen participation and the powerful influence of children’s voices in achieving the goals of Agenda 2030 [51]. SDG 3, Good Health and Well-Being, is the most central goal of the project, as it is well established that physical activity can promote well-being and prevent mental and physical health problems, including obesity. As active transport contributes significantly to daily physical activity [6,7], it is important to encourage children to use active transport and to develop this as a habit from a life-course perspective [21,22]. Given that the intervention is school based and that we integrate educational assignments as teaching sessions and units that teachers can adapt to their class and the level of students, we contribute to SDG 4, Quality Education for All, regardless of gender and socioeconomic background [51]. As the assignments are based on the subject curricula, the children are given opportunities to learn on the way to school, and students can transfer knowledge to reality. In summary, early investments in children’s health, education, and development have benefits across the life course, extending to future generations and society.

### Conclusions

The school-based intervention SICTA is a unique intervention that targets children’s transportation modes to improve health and reduce climate impact by increasing AST. By using an age-cohort design to assess this complex intervention within a dynamic school setting, the findings are expected to provide valuable and novel insights into the effectiveness and sustainable value of the SICTA intervention, facilitating knowledge dissemination among end users and policymakers.

## Appendix

Multimedia Appendix 1 Overview of data collection at baseline and follow-up.

Multimedia Appendix 2 Peer-reviewer report from the Swedish Research Council for Health, Working Life and Welfare (FORTE).

## Abbreviations

AST: active school transportation
BRACS: behavioral regulation in active commuting to and from school
CIM: children’s independent mobility
PILCAST: parents’ intention to let their child use active school transportation
RCT: randomized controlled trial
SDG: Sustainable Development Goal
SICTA: Sustainable Innovation for Children Transporting Actively
