# Supplementary material for: Evaluating the Effectiveness of the School-Based Sustainable Innovation for Children Transporting Actively Intervention: Protocol for an Age-Cohort Study
Source: JMIR Res Protoc. 2026 Jun 5;15:e92946. doi: 10.2196/92946 (PMC13282597; doi:10.2196/92946)
Supplement: Multimedia Appendix 1 [file resprot_v15i1e92946_app1.docx]

| **Appendix 1.** Overview of the data collection at baseline and follow up | |  |  | |  |
| --- | --- | --- | --- | --- | --- |
| **Outcomes** | **Participant** | **Data source** | **Measurement timepoints** | | **Research question** |
|  |  |  | **Baseline (T_0_)** | **Follow-up (T_1_)** |  |
| No. active school transport  Transportation mode  Transportation time  Transportation distance | Children | Web-form: wASTapp | X | X | 1 |
| Child Independent Mobility | Children | Questionnaire: CIM License | X | X | 2 |
| Intrinsic motivation  Integrated regulation  Identified regulation  Introjected regulation  External regulation  Amotivation | Children | Questionnaire: BRACS-SWE | X | X | 3 |
| Intention  Attitudes  Social norm  Perceived behavioral control | Parents | Questionnaire: PILCAST | X | X | 3 |
| Sustainability impact including feasibility evaluation (environmental-, social-, financial impact) | Children | Web-form: wASTapp  Questionnaire : BRACS-SWE  Questionnaire : CIM-License | X | X | 4 |
|  | Parents | Questionnaire: PILCAST | X | X |  |
|  | Teachers | Questionnaire: Feasibility (demand, acceptability, implementation, practicality, limited efficacy) |  | X |  |
| Note: wASTapp = Web-form to assess daily active school transportation, CIM License = Children’s Independent Mobility License, BRACS-SWE = Behavioral regulation in active commuting to and from school questionnaire in Sweden, PILCAST= Parents intention to let their child use active school transportation | | | | | |
