# Supplementary material for: Evaluating the Effectiveness of the School-Based Sustainable Innovation for Children Transporting Actively Intervention: Protocol for an Age-Cohort Study
Source: JMIR Res Protoc. 2026 Jun 5;15:e92946. doi: 10.2196/92946 (PMC13282597; doi:10.2196/92946)
Supplement: Multimedia Appendix 2 [file resprot_v15i1e92946_app2.pdf]

FORTE: Forskningsrådet för hälsa, arbetsliv och välfärd

Beredningsgruppens yttrande

**2023-00484**	**Annie Palstam**	**Beredningsgrupp: HB 2023**

**Utlysningsnamn:** Forskningsprojekt 2023	**Bidragsform:** Projektbidrag
**Projekttitel (svenska):** Effektutvärdering av den skolbaserade interventionen Sustainable Innovation for Children Transporting Actively (SICTA)	**Sökt inriktning:** Fritt

Bedömning

Generella krav

The project is relevant to Forte's areas of responsibility with its focus on increasing physical activity (PA) among children through an intervention aimed at active school transport (AST). Gender perspectives are going to be considered in the project, and ethical issues are well reflected.

Vetenskaplig kvalitet

The aims of the project are formulated in a precise way. The background and literature review are well described, with sound arguments for why AST may contribute to reverse the negative trend in PA and related negative health outcomes among children. The proposed Sustainable Innovation for Children Transporting Actively (SICTA) intervention is well described and seems firmly grounded in social cognitive theory and principles of empowerment and gamification. The applicants offer sound and insightful reflections on the experiences from the pilot of the SICTA intervention, indicating that the intervention was perceived as fun by the children, safe by parents and simple by teachers. The approach to intervention research is clearly stated, and the scaling up of the intervention seems to be vital in establishing the effects. In an innovative way, the applicants suggest using principles for sustainable healthcare and the value equation to evaluate the intervention.
The use of age-cohort design is well substantiated, and the applicants seem to be aware of both the strengths and weaknesses of such a design. The power calculations seem sensible and reasonable, albeit the possibility of design effects is not mentioned. Data collection on number of active school transports by an app developed for the study (the wASTapp) is feasible and likely to generate valid and reliable data. Questionnaires seem to be based on sound theoretical approaches such as the theory of planned behaviour, self-determination theory and the transtheoretical model of change. The triple bottom line equation is an innovative way to evaluate the sustainability of the intervention, including assessment of environmental, social and financial impacts. One concern is that one of the potential obstacles for AST, namely traffic safety, has received too little attention in the application.
This is an innovative and original project. The research group mainly includes scholars from physiotherapy, indicating a low potential for multidisciplinary collaboration.

Samhällsrelevans och nyttiggörande

If the intervention turns out to be effective in increasing AST, the project has a clear societal impact by improving physical and mental health of children. One of the strengths of the project is that students and teachers are actively involved in the project. The plans for utilization and communication of results are described in a clear and good way.

Genomförbarhet

The work plan seems feasible and realistic. The main applicant has published several articles in international peer reviewed journals, but primarily on research in physiotherapy or PA with elderly patients. The other participants in the research group have good and relevant publication records. The budget seems reasonable.

Sammanvägd bedömning

The strengths of the application are its very clear public health relevance, appropriate theoretical approach, feasible plan for implementation and continued development of the intervention and data collections, as well as user participation. However, concerns about traffic safety could have been discussed further. The project is original in its use of principles for sustainable healthcare and the value equation to evaluate the intervention.

Förslag till beslut (bevilja, reserv, avslå)

Bevilja
